# Assessment of the (Pro)renin Receptor Protein Expression in Organs

**DOI:** 10.3390/cimb46030113

**Published:** 2024-02-25

**Authors:** Teng-Yao Yang, Pey-Jium Chang, Yu-Shien Ko, Siou-Ru Shen, Shun-Fu Chang

**Affiliations:** 1 Cardiovascular Department, Chiayi Chang Gung Memorial Hospital, Chiayi 613, Taiwan; yaoyao1004@gmail.com; 2 Graduate Institute of Clinical Medical Sciences, College of Medicine, Chang Gung University, Taoyuan 333, Taiwan; peyjiumc@mail.cgu.edu.tw; 3 Cardiovascular Division, Department of Internal Medicine, Linkou Chang Gung Memorial Hospital, Taoyuan 333, Taiwan; c12037@adm.cgmh.org.tw; 4 College of Medicine, Chang Gung University, Taoyuan 333, Taiwan; 5 Department of Medical Research and Development, Chiayi Chang Gung Memorial Hospital, Chiayi 613, Taiwan; 6 Center for General Education, Chiayi Chang Gung University of Science and Technology, Chiayi 613, Taiwan

**Keywords:** cardiovascular disease, M8.9 fragment, (pro)renin receptor, renin-angiotensin system, s(P)RR

## Abstract

The (pro)renin receptor ((P)RR) is an essential component of the renin–angiotensin system (RAS) as a specific single-pass transmembrane receptor for prorenin and renin and has now emerged as a multifunctional protein implicated in a wide variety of developmental and physio-pathological processes and pathways. The (P)RR may be of pathological significance in metabolic syndrome. The (P)RR has received much consideration; substantial efforts have been made to understand the localization, regulation, and function of the (P)RR at both a molecular and system level. (P)RR regulation of cell function depends on whether it is intact or cleaved into its constituent forms. Therefore, the present chapter describes immunohistochemical approaches to examine the expression of (P)RR in various organs. It was shown that different molecular forms of (P)RR could be present in different tissue compartments in almost all organs. Among them, the liver has high PRR activity. Our findings could elucidate more detailed distribution of different (P)RR molecular forms in different organs, which could provide useful information to further investigate the pathophysiological mechanisms of the development of various diseases in the future.

## 1. Introduction

(Pro)renin receptor ((P)RR), also nominated as ATP6AP2, is a type I single-pass transmembrane protein consisting of 350 amino acids with a long extracellular domain, a transmembrane domain, and a short intracellular domain [1,2]. In 2002, (P)RR was first identified as a receptor of the renin–angiotensin system (RAS), in which (pro)renin binds to (P)RR and then enhances the cleavage of angiotensinogen (AGT) to angiotensin (Ang) I; subsequently, Ang I could be further converted to Ang II [2]. Because RAS has been elucidated as important in controlling vessel pressure/resistance and salt/water reabsorption of the kidney, evidence has also confirmed the pathogenic role of (P)RR in vascular and kidney-related diseases [1,3,4]. Moreover, it has been further suggested that the discovery of (P)RR might provide a plausible rationale for an issue about why a high Ang II activity could be caused by an extreme low expression level of renin in some tissues [5,6]. After two decades of study, (P)RR has been shown to widely express in many types of organs/tissues, e.g., vessel, heart, thyroid, brain, kidney, liver, colon, etc. [4,7]. In addition, the pathogenic role and mechanism of (P)RR in various diseases, e.g., heart and kidney failure, hypertension, diabetes, obesity, and cancers, have also been further proposed and elucidated [4]. However, the exact role of (P)RR in different organs/tissues still needs more investigation and elucidation because of its ubiquitous expression throughout the body, high clinical pathogenic correlation, and complicated multifaceted mechanism.

In addition to being the receptor to (pro)renin, it has been found that full-length (P)RR (f(P)RR) might be cleaved by different proteases in different environments to produce a soluble form of (P)RR (s(P)RR), which is the extracellular domain of f(P)RR and a residual fragment which contains the transmembrane and cytoplasmic domains [8]. In 2009, furin was the first protease found to cleave (P)RR at the R^275^KTR^278^ amino acid sites in the trans-Golgi [9,10]. Two years later, a disintegrin and metalloproteinase 19 (ADAM19) was also shown to cleave (P)RR, but it was processed in the Golgi apparatus and in a furin-independent manner [10]. In 2017, site-1 protease (S1P) was indicated as another protease cleaved (P)RR at R^278^TIL^281^ amino acid sites; in addition, it was suggested that S1P and furin might cleave (P)RR through sequential processing [11]. Recently, a convertase was further proposed to promote s(P)RR production in the placenta, which is furin- and S1P-independent, but the underlying mechanism is still unclear [12]. Although these complicated findings lead to a still unsolved (P)RR cleavage mechanism elucidation, accumulating data have already evidenced the importance of s(P)RR production. After cleavage, s(P)RR could be released into the extracellular fluid, such as plasma and urine [9,10,11,12]. Similar to the regulatory mission of f(P)RR, s(P)RR has also confirmed its role in controlling RAS activation, vessel function, and electrolyte balance and confirmed its clinical correlation in the development of various diseases [13,14,15,16,17]. Furthermore, although it still has controversy, the residual transmembrane and intracellular domains after (P)RR cleavage might be defined as M8.9 fragment. There is evidence that M8.9 fragment might be an accessory member of vacuolar H^+^-ATPase (V-ATPase) [6,18].

In addition to participating in RAS activation, (P)RR could also directly initiate downstream signaling, independent of Ang II, to modulate the cell functions and hence affect pathogenic development of myocardium, kidney, and many other organs/tissues. These signaling pathways include mitogen-activated protein kinases (MAPKs), Wnt/β-catenin, and V-ATPase [6]. After binding with (pro)renin, (P)RR could activate MAPKs signaling, including the extracellular signal-related protein kinase (ERK) 1/2, p38, or phosphatidylinositol 3-kinase (PI3K) pathways to modulate many types of cell functions, such as survival, proliferation, oxidative stress, inflammation, fibrosis, and so on [6]. Moreover, (P)RR could also crosslink with Wnt/β-catenin and V-ATPase to control the target gene expression and influence the intracellular microenvironment, respectively [6,19,20]. In addition, it has also been shown that the hydrolase activity of lysosomes could also be regulated by crosslinking between the M8.9 fragment and V-ATPase [6].

Taken together, (P)RR might be a multifunctional protein and its complex regulatory mechanisms might be activated in a context-dependent manner, which includes that different mechanisms could be initiated by different (P)RR molecular forms, i.e., f(P)RR, s(P)RR, and M8.9 fragment, in different organs/tissues. In the present study, the localization of different molecular forms of (P)RR was analyzed and the results showed that different molecular forms of (P)RR might be expressed in different tissue compartments in the kidney, pancreas, heart, and brain but not in the liver. Our findings could elucidate more detailed distribution of different (P)RR molecular forms in different organs, which could provide useful information to further investigate the pathophysiological mechanisms of the development of various diseases in the future.

## 2. Materials and Methods

### 2.1. Materials

Human embryonic kidney cells 293T (HEK293T) were purchased from the Cell Bank of the American Type Culture Collection (Rockville, MD, USA). Specific antibody for anti-N-terminal (P)RR (anti-N-(P)RR) was purchased from the Abcam (ab64957, Cambridge, UK). Specific antibody for anti-C-terminal (P)RR (anti-C-(P)RR) was purchased from the Everest biotech (EB06118, Oxfordshire, UK). Restriction enzymes, T4 DNA ligase, lipofectamine 2000 reagent, and lysis buffer were purchased from Thermo (Waltham, MA, USA). Other materials that are not described were purchased from Sigma-Aldrich (St. Louis, MO, USA).

### 2.2. Cell Culture

HEK293T cells were kept in Dulbecco’s Modified Eagle Medium (DMEM) with serum (10%) and antibiotics (1%) and were incubated in an incubator with 37 °C and 5% CO_2_ conditions. The materials used in culturing the cells were purchased from Thermo (Waltham, MA, USA).

### 2.3. Construction of (P)RR-Overexpressed Plasmid

**Figure 1:**
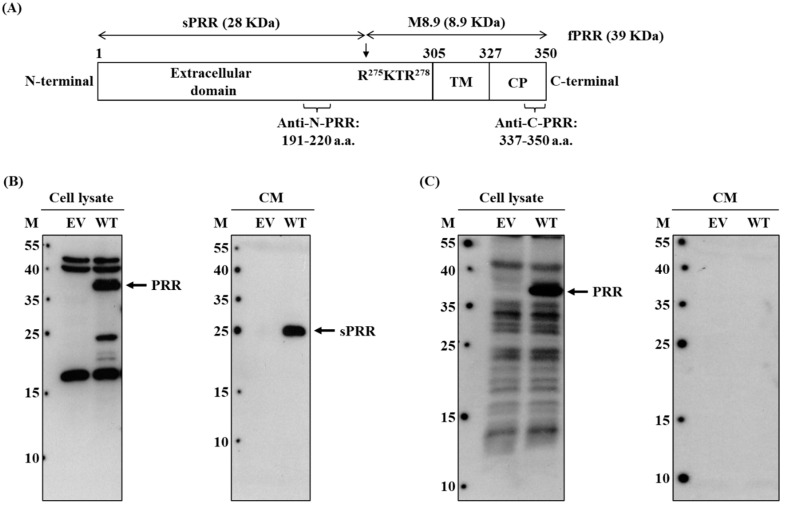
Specificity validation of anti-N-(P)RR and ani-C-(P)RR antibodies. (**A**) The diagram of f(P)RR with different domains and the antigen sequences of anti-N-(P)RR and anti-C-(P)RR antibodies. TM: transmembrane domain, CP: cytoplasmic domain, a.a.: amino acid. (**B**,**C**) Cells were transfected with empty vector (EV) or (P)RR wild-type (WT)-overexpressed plasmid for 48 h and then the cell lysate and cell-culturing conditional medium (CM) were collected to examine the expression or secretion levels of different molecular forms of (P)RR by using the Western blot with anti-N-(P)RR (**B**) and anti-C-(P)RR antibodies (**C**). The experiments were repeated independently at least three times.

The DNA fragment of (P)RR wild type (Figure 1A) was linked into the pCMV-3Tag-3 plasmid (Addgene, Watertown, MA, USA) by the EcoRI and Xho I restriction enzymes and T4 DNA ligase. The DNA fragment of (P)RR wild type was amplified by the primers: (forward) 5′-CGT GAA TTC ACC ATG GCT GTG CTT GTC GTT C-3′ and (reverse) 5′-GGA CTC GAG ATC CAT TCG AAT CTT CTG G-3′.

### 2.4. Transfection of (P)RR-Overexpressed Plasmid

HEK293T cells (60–70% confluence) were cultured in 3.5 cm dishes with antibiotic-free DMEM medium for 24 h before transfection. Transient transfection with plasmid pCMV-3Tag-3 empty vector (EV) and pCMV-3Tag-3-(P)RR wild type was performed 24 h after plating using Lipofectamin2000 reagent (Invitrogen, Carlsbad, CA, USA) according to the manufacturer’s instructions. A total of 5 µg of deoxyribonucleic acid and 15 µL of Lipofectamine2000 reagent were each diluted into 250 µL Opti-MEM (Invitrogen, Carlsbad, CA, USA), mixed, and added to cells cultured in 3.5 cm dishes. After 4 h, the cells were washed and cultured in fresh medium for 4 h. Subsequently, the cells were plated onto 10 cm dishes for Western blotting.

### 2.5. Western Blot Analysis

After 48 h transfection, the transfected cells were collected and the total proteins were extracted from cells by adding the commercial lysis buffer. Protein concentration was measured by using the Bio-Rad protein assay kit (Bio-Rad, Hercules, CA, USA). A total of 150 μg of protein extract was separated using sodium dodecyl sulfate-polyacrylamide gel electrophoresis and then transferred onto a polyvinyl difluoride membrane with 0.45 µm pore size (Bio-Rad, Hercules, CA, USA). After that, the membrane was blocked by using 5% milk and subsequently incubated with the anti-N-(P)RR and anti-C-(P)RR primary antibodies and the horse-radish peroxidase-conjugated secondary antibody. Immunoreactive bands were visualized by using the enhanced chemiluminescence detection system (Applied Biosystems, Foster, CA, USA).

### 2.6. Animals

All the animal experiments and protocols were approved by the Institutional Animal Care and Use Committee (No. 2019122304) in Chiayi Chang Gung Memorial Hospital. Sprague–Dawley (SD) rats (male, ight weeks old) were purchased from the BioLASCO Taiwan Co., Ltd. (Taipei, Taiwan). Rats were housed in a suitable and controlled environment (temperature: 23 ± 2 °C; relative humidity: 50 ± 5%) with a 12 h light/dark cycle and were allowed free access to food and water ad libitum. The organs, including kidney, liver, pancreas, heart, and brain, were excised after rats were anesthetized with isoflurane inhalation. These organs were further analyzed by using immunohistochemical staining assay.

### 2.7. Immunohistochemical Staining

Organs were embedded in Tissue-Tek OCT (optimal cutting temperature) compound (Sakura Finetek, Torrance, CA, USA), frozen, and sectioned into 12–16 µm cryosections. Cryosections were fixed with 10% neutral buffered formalin for 15 min at room temperature and washed 3 times with PBS. Cryostat sections were incubated with 0.1% TritonX-100 for 15 min at room temperature and washed 3 times with PBS. To block unspecific binding of antibodies, cryosections were incubated with 1% BSA in PBS for 30 min prior to the primary antibody. The primary antibodies (rabbit polyclonal anti-N-(P)RR 1:500 and goat anti-C-(P)RR 1:500) were diluted in PBS and applied for overnight at 4 °C. Cryosections were then washed 3 times with PBS and incubated with secondary donkey anti-rabbit Alexa fluor 488 (1:50, Invitrogen, Carlsbad, CA, USA) and/or donkey anti-goat Alexa fluor 594 (1:50, Invitrogen, Carlsbad, CA) antibodies for 60 min. Nuclei were stained blue with 4,6-diamidino-2-phenylinodole (DAPI, Invitrogen, Carlsbad, CA, USA) for 15 min at room temperature. Cryosections were again washed 3 times with PBS and once with PBS before mounting with glycergel mounting medium (Dako, Carpinetria, CA, USA) and were sealed with a coverslip. Cryosections were viewed with a Leica TCS SP5 II confocal microscope (Leica Microsystems, Wetzlar, Germany) and, for images comparing localization and intensity of staining, pictures were taken on the same day and with identical settings for gain, intensity, and fluorescence filters. Images were processed using Image J.

## 3. Results

### 3.1. Specificity Validation of anti-N-(P)RR and anti-C-(P)RR Antibodies

To investigate the exact localization of different molecular forms of (P)RR, i.e., f(P)RR, s(P)RR, and M8.9 fragment, in different organs, the specificity of commercial anti-N-(P)RR antibody, which recognizes the amino acid sequences of (P)RR from 191th to 220th, and anti-C-(P)RR antibody, which recognizes the amino acid sequences of (P)RR from 337th to 350th, should be evidenced firstly (Figure 1A). HEK293T cells were transfected with empty vector (EV) or (P)RR wild-type (WT)-overexpressed plasmid for 48 h and then the cell lysate and cell-culturing conditional medium (CM) were collected to examine the expression or secretion levels of different molecular forms of (P)RR by using the Western blot with both commercial antibodies. It was shown that anti-N-(P)RR antibody could identify the f(P)RR expression (left panel of Figure 1B) and s(P)RR secretion (right panel of Figure 1B) levels in the cell lysate and conditional medium, respectively, in the (P)RR-WT-overexpressed cells compared to those of the EV-transfected cells. Anti-C-(P)RR antibody could also identify the expression level of f(P)RR in the cell lysate of the (P)RR-WT-overexpressed cells compared to that of the EV-transfected cells (left panel of Figure 1C). However, it could not detect the secretion level of s(P)RR in the conditional medium of both EV-transfected and (P)RR-WT-overexpressed cells (right panel of Figure 1C). These results confirmed the specificity of both commercial antibodies in detecting the expression and secretion levels of f(P)RR and s(P)RR, respectively.

### 3.2. Different Localization of f(P)RR, s(P)RR, and M8.9 Fragment in the Kidney

**Figure 2:**
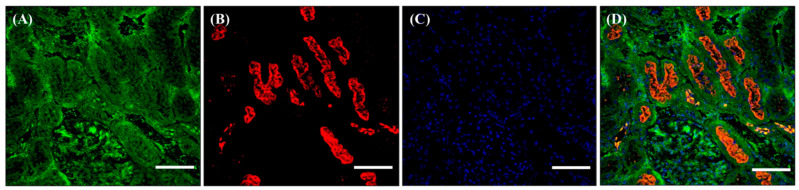
Different localization of f(P)RR, s(P)RR, and M8.9 fragment in the kidney. The kidney was collected from the SD rats and the exact localization of f(P)RR, s(P)RR, and M8.9 fragment in the kidney was examined by using immunohistochemical staining with anti-N-(P)RR (**A**, green) and anti-C-(P)RR antibodies (**B**, red). (**C**) The cell nucleus was stained by DAPI reagent (blue). (**D**) The merge image indicates the distribution and overlapping of different molecular forms of (P)RR and nuclei. Scale bar: 75 mm.

As described in the Materials and Methods, the organs were collected from the SD rats and the exact localization of f(P)RR, s(P)RR, and M8.9 fragment was examined by using the immunohistochemical staining with both commercial antibodies. Within the kidney, it was shown that s(P)RR could localize in all tissues (Figure 2A,D), but f(P)RR and M8.9 fragment could only be found in the lumen of renal tubules (Figure 2B,D). This is because signals positive for anti-N-(P)RR antibody, indicating the location of f(P)RR and/or s(P)RR, were found in all kidney tissues, including mesangium, Bowman’s capsule, and lumen of renal tubules (Figure 2A,D). However, signals positive for anti-C-(P)RR antibody, indicating the location of f(P)RR and/or M8.9 fragment, were only found in the lumen of renal tubules (Figure 2B,D).

### 3.3. Different Localization of f(P)RR, s(P)RR, and M8.9 Fragment in the Liver

**Figure 3:**
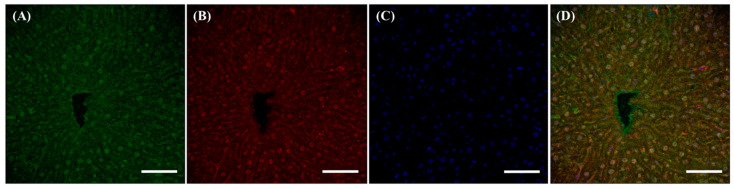
Different localization of f(P)RR, s(P)RR, and M8.9 fragment in the liver. The liver was collected from the SD rats and the exact localization of f(P)RR, s(P)RR, and M8.9 fragment in the liver was examined by using immunohistochemical staining with anti-N-(P)RR (**A**, green) and anti-C-(P)RR antibodies (**B**, red). (**C**) The cell nucleus was stained by DAPI reagent (blue). (**D**) The merge image indicates the distribution and overlapping of different molecular forms of (P)RR and nuclei. Scale bar: 75 mm.

Within the liver, no significant differences in the localization of f(P)RR, s(P)RR, and M8.9 fragment were found, as signals positive for anti-N-(P)RR and anti-C-(P)RR antibodies overlapped in almost all tissues (Figure 3).

### 3.4. Different Localization of f(P)RR, s(P)RR, and M8.9 fragment in The Pancreas

**Figure 4:**
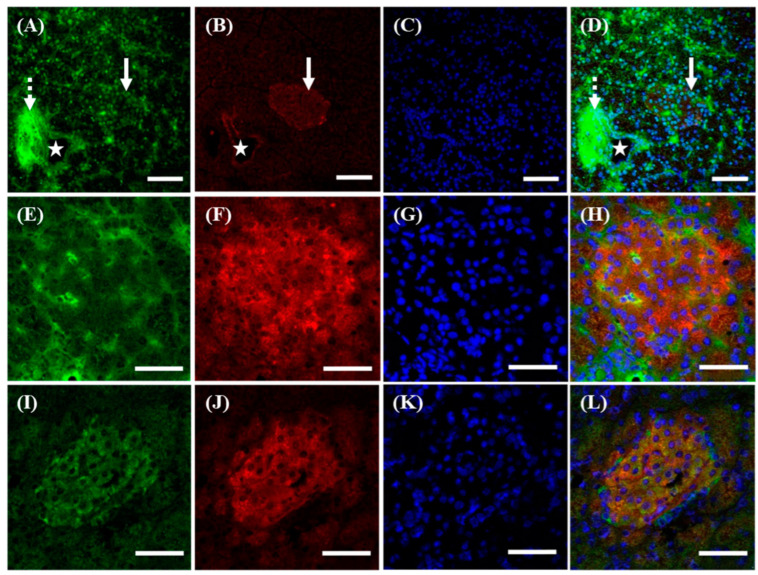
Different localization of f(P)RR, s(P)RR, and M8.9 fragment in the pancreas. The pancreas was collected from the SD rats and the exact localization of f(P)RR, s(P)RR, and M8.9 fragment in the pancreas was examined by using immunohistochemical staining with anti-N-(P)RR (**A**, green) and anti-C-(P)RR antibodies (**B**, red). (**E**,**F** and **I**–**L**) The co-localization of f(P)RR, s(P)RR, and M8.9 fragment with insulin was examined by using immunohistochemical staining with anti-N-(P)RR (**E**, green), anti-C-(P)RR (**I**, green), and anti-insulin (**F**,**J**, red) antibodies. (**C**,**G**,**K**) The cell nucleus was stained by DAPI reagent (blue). (**D**,**H**,**L**) The merge image indicates the distribution and overlapping of different molecular forms of (P)RR, insulin, and nuclei. Star indicates vessel; dotted arrow indicates exocrine duct system; solid arrow indicates islets of Langerhans. Scale bar in (**A**–**D**): 75 mm. Scale bar in (**E**–**L**): 50 mm.

Within the pancreas, it was shown that s(P)RR, signals positive for anti-N-(P)RR antibody, could localize in all tissues, including the vessel wall (marked by a star, Figure 4A,D), exocrine duct system (marked by a dotted arrow, Figure 4A,D), and islets of Langerhans (marked by a solid arrow, Figure 4A,D). However, f(P)RR and M8.9 fragment, signals positive for anti-C-(P)RR antibody, could only be found in the vessel wall (marked by a star, Figure 4B,D) and islets of Langerhans (marked by a solid arrow, Figure 4B,D). To further analyze if the distribution of different molecular forms of (P)RR was associated with insulin-producing β-cells in the islets of Langerhans, the co-localization of f(P)RR, s(P)RR, and M8.9 fragment with insulin was examined by using immunohistochemical staining with anti-N-(P)RR (Figure 4E), anti-C-(P)RR (Figure 4I), and anti-insulin (Figure 4F,J) antibodies. It was indicated that all types of (P)RR might be present in the cytoplasmic and peri-nuclear regions of β-cells (Figure 4E,H,I,L), as they were found to co-localize with insulin (Figure 4F,H,J,L) in the islets of Langerhans.

### 3.5. Different Localization of f(P)RR, s(P)RR, and M8.9 Fragment in the Heart

**Figure 5:**
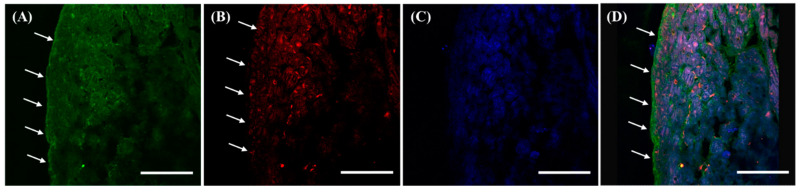
Different localization of f(P)RR, s(P)RR, and M8.9 fragment in the heart. The heart was collected from the SD rats and the exact localization of f(P)RR, s(P)RR, and M8.9 fragment in the heart was examined by using immunohistochemical staining with anti-N-(P)RR (**A**, green) and anti-C-(P)RR antibodies (**B**, red). (**C**) The cell nucleus was stained by DAPI reagent (blue). (**D**) The merge image indicates the distribution and overlapping of different molecular forms of (P)RR and nuclei. Solid arrow indicates endocardium. Scale bar: 50 mm.

Within the heart, different molecular forms of (P)RR were shown in almost all cardiac tissues except endocardium (marked by a solid arrow), as positive signals from both anti-N-(P)RR and anti-C-(P)RR antibodies were present in those regions (Figure 5A–D). However, in the endocardium, it was indicated that only s(P)RR was present there, as only anti-N-(P)RR antibody showed positive signals there (marked by an arrow, Figure 5A,D).

### 3.6. Different Localization of f(P)RR, s(P)RR, and M8.9 Fragment in the Brain

**Figure 6:**
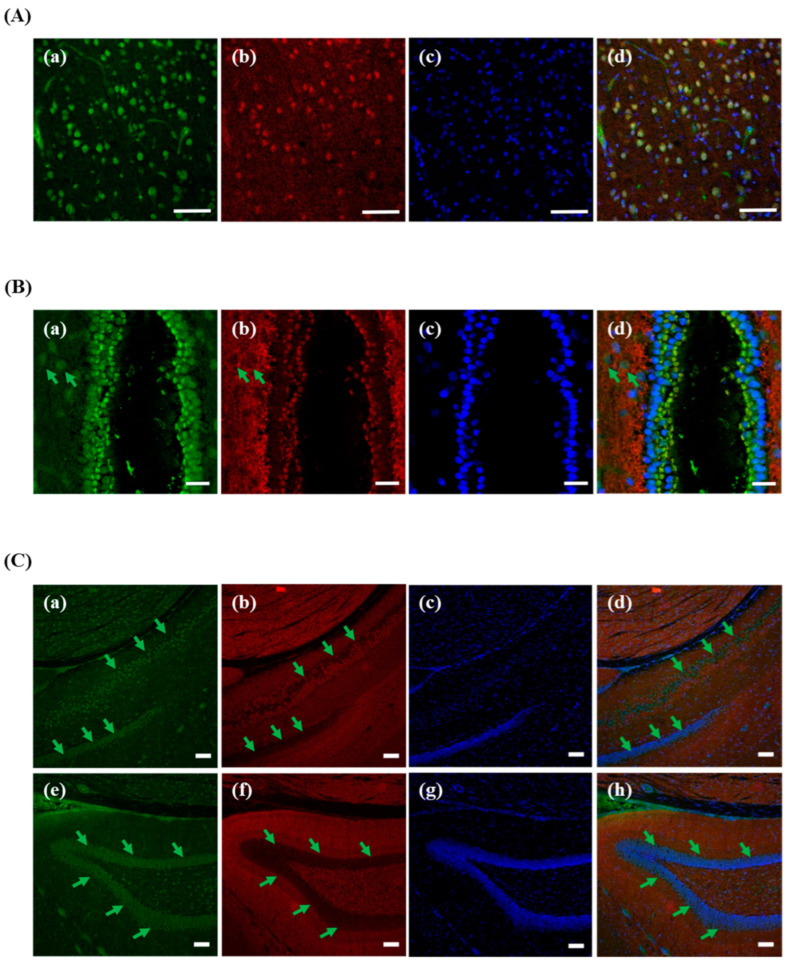
Different localization of f(P)RR, s(P)RR, and M8.9 fragment in the brain. The brain was collected from the SD rats and the exact localization of f(P)RR, s(P)RR, and M8.9 fragment in brain cortex (**A**), third ventricle (**B**), and hippocampus (**C**) was examined by using immunohistochemical staining with anti-N-(P)RR (a in **A**–**C** and e in **A**–**C**, green) and anti-C-(P)RR antibodies (b in **A**–**C** and f in **C**, red). (**A**–**C**) The cell nucleus was stained by DAPI reagent (c in **A**–**C** and g in **C**, blue). (**A**–**C**) The merge image indicates the distribution and overlapping of different molecular forms of (P)RR and nuclei (d in **A**–**C** and h in **C**). Green arrow indicates (**B**) tanycytes and (**C**) radiatum layer and dentate gyrus. Scale bar is 75 mm in (**A**); 25 mm in (**B**); and 100 mm in (**C**).

Finally, different molecular forms of (P)RR in the brain cortex (Figure 6A), third ventricle (Figure 6B), and hippocampus (Figure 6C) were analyzed. It was shown that all types of (P)RR were found in the neurons of the brain cortex (Figure 6A(a–d)).The more interesting findings in the third ventricle of brain showed that s(P)RR is mainly localized in the tanycytes (marked by a green arrow, Figure 6B(a,d), but M8.9 fragment is mainly localized in the arcuate nucleus and dorsomedial hypothalamic nucleus areas (Figure 6B(b,d)). Moreover, within the hippocampus, s(P)RR and M8.9 fragment were also found to localize in different hippocampus layers: s(P)RR is mainly localized in the radiatum layer and dentate gyrus (marked by a green arrow, Figure 6C(a,d,e,h) and M8.9 fragment is mainly localized in the cornu ammonis subfields (Figure 6C(b,d,f,h)).

## 4. Discussion

(P)RR has been indicated as a context-dependent regulatory receptor because it has been found that (P)RR in different organs/tissues might have various effects. This study examined the localization of different molecular forms of (P)RR, including s(P)RR, f(P)RR, and M8.9 fragment, in multiple organs, i.e., kidney, liver, pancreas, heart, and brain, and found that different molecular forms of (P)RR might exist in different tissue compartments in most of the organs except the liver: (i) s(P)RR could localize in all tissue compartments of the kidney but f(P)RR and M8.9 fragment could only co-localize in the renal tubules; (ii) s(P)RR could localize in all tissue compartments of the pancreas but f(P)RR and M8.9 fragment could only co-localize in the vessel wall and islets of Langerhans; (iii) all types of (P)RR could co-localize in all cardiac tissue compartments except the endocardium, where only s(P)RR was present; and (iv) all types of (P)RR could localize in all tissue compartments of the brain cortex; moreover, s(P)RR could localize in the tanycytes of the third ventricle of brain as well as in the radiatum layer and dentate gyrus of the hippocampus but M8.9 fragment could localize in the arcuate nucleus and dorsomedial hypothalamic nucleus areas of the brain third ventricle as well as in the cornu ammonis subfields of the hippocampus.

The distribution of (P)RR in the kidney has still remained controversial. However, most of the studies have evidenced the abundance of (P)RR in the renal tubules [21], which also supports our finding about the co-localization of f(P)RR and M8.9 fragment in the renal tubules. (P)RR is essential to nephron development/function and blood pressure control through regulating the lysosomal acidification and sodium/water balance under physiological conditions [21]. The pathogenic role of (P)RR in hypertension and kidney injury have been extensively studied and evidenced. However, the discovery of s(P)RR has provided a more complex effect and mechanism for the development of related diseases [15,22,23,24]. In addition to co-localizing in renal tubules, our study also showed that s(P)RR could exist in almost all tissue compartments of the kidney. Recently, it has been indicated that the reduction in s(P)RR level might alleviate Ang-II-initiated hypertension and renal injury [24]. Moreover, more studies have further elucidated the pathogenic role of s(P)RR in controlling sodium/water balance, blood pressure, and kidney injury [25,26,27,28]. Therefore, our finding of ubiquitous s(P)RR in all tissue compartments of the kidney could confirm the importance of s(P)RR in the kidney and raise the need for further research.

Our data showed that all types of (P)RR co-localize in the hepatocytes with similar subcellular location. A novel, Ang-II-independent pathway of (P)RR is a regulatory mechanism of lipoprotein metabolism. The liver is the main organ in controlling the regulation of low-density lipoprotein (LDL). Recently, sortilin-1 has been identified to be a hepatic clearance receptor for LDL and also regulate the LDL metabolism, very low-density lipoprotein (VLDL) secretion, and plasma triglycerides [29,30,31]. Moreover, it has been further indicated that (P)RR could be a sortilin-1-interacting protein and the silence of (P)RR gene expression in hepatocytes could reduce the protein abundance of sortilin-1 and consequent post-transcriptional mechanism of LDLR and cellular LDL uptake [30]. Therefore, the role of (P)RR in lipid metabolism and liver function is an interesting and important issue for further investigation.

Previous studies have shown that (P)RR might participate in the pathogenesis of glucose intolerance, at least in part, through RAS-dependent mechanisms [32,33,34]. Moreover, it has been further indicated that (P)RR could be found in both alpha and beta cells in the islets of Langerhans to modulate glucagon-like peptide 1 (GLP1) receptor (GLP1R) signaling and insulin processing and hence controls insulin secretion [34,35,36]. GLP1 is an incretin hormone that enhances insulin secretion by interacting and activating its receptor, GLP1R. Furthermore, it has also been found that (P)RR levels would be reduced in the islets of Langerhans of patients with diabetes mellitus compared to healthy subjects [36]. All of these data support our findings that all types of (P)RR could exist in the islets of Langerhans and co-localize with insulin. This evidence, including ours, could demonstrate the role of (P)RR in controlling the insulin metabolism and subsequent blood glucose homeostasis.

Our results showed that all types of (P)RR were found in almost all tissue compartments of the heart except the endocardium, where only s(P)RR was present. The role of (P)RR in cardiac disease development through RAS-dependent and RAS-independent pathway have been evidenced. It has been further elucidated that (P)RR activation in myocardium is associated with the development of cardiac hypertension/fibrosis, heart failure, diabetic cardiomyopathy, and ischemia/reperfusion damage [37]. The endocardium is the innermost soft layer of the heart that covers the chambers’ inner surface and forms the valves’ surface, protecting these tissues from damage. Two of the important roles of endocardium are to control myocardium contraction through paracrine mechanisms and to form a kind of blood–heart barrier controlling ion homeostasis. Moreover, in cardiac pathogenesis, including heart failure and ischemia/reperfusion damage, dysfunctional endocardium and myocardium have further shown their reciprocal paracrine regulation to promote disease progression [38,39]. All of this evidence illuminates the importance of endocardial–myocardial crosstalk in controlling the physiological and pathophysiological situations of cardiac tissues. Therefore, our finding regarding the unique presence of s(P)RR in the endocardium suggested a possible role for s(P)RR in regulating the communication between the endocardium and myocardium.

RAS could exist in many organs/tissues to play an important regulating role in controlling blood pressure. Renin is the most critical component to enhance RAS activation [5,6,40]. However, early on, an interesting phenomenon has been found in many organs/tissues, including the brain, where renin expression is extremely low but Ang-II activity is high. Moreover, it has been further shown that these organs/tissues might express high prorenin, which is only ~3% as active as renin; the enzymatic cleavage of prorenin into renin does not occur [40]. This confusing phenomenon could not be reasonably explained until the discovery of (P)RR. The binding of prorenin with (P)RR could significantly increase prorenin activity to initiate the RAS mechanism [5,6,40]. In our study, although there was no difference in the distribution of all types of (P)RR in the brain cortex, the distribution of s(P)RR and M8.9 fragment in the third ventricle and hippocampus showed obvious differences. These intriguing findings suggest that RAS components, including different molecular forms of (P)RR, in the brain might have more complex mechanisms to control blood pressure or other pathophysiological events.

Limitations: there are some limitations to this study. First, this study only analyzed normal tissue and lacked pathological tissue results, making it impossible to verify the physiological and pathophysiological roles of different forms of (P)RR. Second, the functional roles of (P)RR could not be determined by this immunohistochemical study. However, this study demonstrated that different types of (P)RR might have different tissue compartment distributions in the analyzed organs (except the liver), providing important information on the roles of (P)RR in the development of various diseases.

## 5. Conclusions

In the present study, different molecular forms of (P)RR were examined in various organs. Our results showed that different types of (P)RR might have different tissue compartment distributions in the analyzed organs (except the liver). These findings suggested that the current mechanism elucidation of (P)RR and its subsequent RAS-dependent and RAS-independent signaling requires more precise and detailed investigations.

## Data Availability

Data are contained within the article.

## References

[1] Hoffmann N., Peters J. (2021). Functions of the (pro)renin receptor (Atp6ap2) at molecular and system levels: Pathological implications in hypertension, renal and brain development, inflammation, and fibrosis. Pharmacol. Res..

[2] Nguyen G., Delarue F., Burcklé C., Bouzhir L., Giller T., Sraer J.D. (2002). Pivotal role of the renin/prorenin receptor in angiotensin II production and cellular responses to renin. J. Clin. Investig..

[3] Fyhrquist F., Saijonmaa O. (2008). Renin-angiotensin system revisited. J. Intern. Med..

[4] Ichihara A., Yatabe M.S. (2019). The (pro)renin receptor in health and disease. Nat. Rev. Nephrol..

[5] Chan S.H., Chan J.Y. (2015). (Pro)renin receptor as a therapeutic target for the treatment of hypertension?. Hypertension.

[6] Wang B., Jie H., Wang S., Dong B., Zou Y. (2023). The role of (pro)renin receptor and its soluble form in cardiovascular diseases. Front. Cardiovasc. Med..

[7] Fagerberg L., Hallström B.M., Oksvold P., Kampf C., Djureinovic D., Odeberg J., Habuka M., Tahmasebpoor S., Danielsson A., Edlund K. (2014). Analysis of the human tissue-specific expression by genome-wide integration of transcriptomics and antibody-based proteomics. Mol. Cell. Proteom..

[8] Qin M., Xu C., Yu J. (2021). The Soluble (Pro)Renin Receptor in Health and Diseases: Foe or Friend?. J. Pharmacol. Exp. Ther..

[9] Cousin C., Bracquart D., Contrepas A., Corvol P., Muller L., Nguyen G. (2009). Soluble form of the (pro)renin receptor generated by intracellular cleavage by furin is secreted in plasma. Hypertension.

[10] Yoshikawa A., Aizaki Y., Kusano K., Kishi F., Susumu T., Iida S., Ishiura S., Nishimura S., Shichiri M., Senbonmatsu T. (2011). The (pro)renin receptor is cleaved by ADAM19 in the Golgi leading to its secretion into extracellular space. Hypertens. Res..

[11] Nakagawa T., Suzuki-Nakagawa C., Watanabe A., Asami E., Matsumoto M., Nakano M., Ebihara A., Uddin M.N., Suzuki F. (2017). Site-1 protease is required for the generation of soluble (pro)renin receptor. J. Biochem..

[12] Morosin S.K., Delforce S.J., Lumbers E.R., Pringle K.G. (2020). Cleavage of the soluble (pro)renin receptor (sATP6AP2) in the placenta. Placenta.

[13] Gonzalez A.A., Lara L.S., Luffman C., Seth D.M., Prieto M.C. (2011). Soluble form of the (pro)renin receptor is augmented in the collecting duct and urine of chronic angiotensin II-dependent hypertensive rats. Hypertension.

[14] Lu X., Wang F., Xu C., Soodvilai S., Peng K., Su J., Zhao L., Yang K.T., Feng Y., Zhou S.F. (2016). Soluble (pro)renin receptor via β-catenin enhances urine concentration capability as a target of liver X receptor. Proc. Natl. Acad. Sci. USA.

[15] Yang T. (2022). Potential of soluble (pro)renin receptor in kidney disease: Can it go beyond a biomarker?. Am. J. Physiol. Renal. Physiol..

[16] Zhu Q., Yang T. (2018). Enzymatic sources and physio-pathological functions of soluble (pro)renin receptor. Curr. Opin. Nephrol. Hypertens..

[17] Yang T. (2022). Revisiting the relationship between (Pro)Renin receptor and the intrarenal RAS: Focus on the soluble receptor. Curr. Opin. Nephrol. Hypertens..

[18] Kinouchi K., Ichihara A., Sano M., Sun-Wada G.H., Wada Y., Ochi H., Fukuda T., Bokuda K., Kurosawa H., Yoshida N. (2013). The role of individual domains and the significance of shedding of ATP6AP2/(pro)renin receptor in vacuolar H(+)-ATPase biogenesis. PLoS ONE.

[19] Schafer S.T., Han J., Pena M., von Bohlen Und Halbach O., Peters J., Gage F.H. (2015). The Wnt adaptor protein ATP6AP2 regulates multiple stages of adult hippocampal neurogenesis. J. Neurosci..

[20] Cruciat C.M., Ohkawara B., Acebron S.P., Karaulanov E., Reinhard C., Ingelfinger D., Boutros M., Niehrs C. (2010). Requirement of prorenin receptor and vacuolar H+-ATPase-mediated acidification for Wnt signaling. Science.

[21] Ramkumar N., Kohan D.E. (2016). The nephron (pro)renin receptor: Function and significance. Am. J. Physiol. Ren. Physiol..

[22] Arthur G., Osborn J.L., Yiannikouris F.B. (2021). (Pro)renin receptor in the kidney: Function and significance. Am. J. Physiol. Regul. Integr. Comp. Physiol..

[23] Yang T. (2023). Soluble (Pro)Renin Receptor in Hypertension. Nephron.

[24] Ramkumar N., Stuart D., Peterson C.S., Hu C., Wheatley W., Min Cho J., Symons J.D., Kohan D.E. (2021). Loss of Soluble (Pro)renin Receptor Attenuates Angiotensin-II Induced Hypertension and Renal Injury. Circ. Res..

[25] Yang K.T., Wang F., Lu X., Peng K., Yang T., David Symons J. (2017). The soluble (Pro) renin receptor does not influence lithium-induced diabetes insipidus but does provoke beiging of white adipose tissue in mice. Physiol. Rep..

[26] Gatineau E., Gong M.C., Yiannikouris F. (2019). Soluble Prorenin Receptor Increases Blood Pressure in High Fat-Fed Male Mice. Hypertension.

[27] Feng Y., Peng K., Luo R., Wang F., Yang T. (2021). Site-1 Protease-Derived Soluble (Pro)Renin Receptor Contributes to Angiotensin II-Induced Hypertension in Mice. Hypertension.

[28] Fu Z., Wang F., Liu X., Hu J., Su J., Lu X., Lu A., Cho J.M., Symons J.D., Zou C.J. (2021). Soluble (pro)renin receptor induces endothelial dysfunction and hypertension in mice with diet-induced obesity via activation of angiotensin II type 1 receptor. Clin. Sci..

[29] Su X., Peng D. (2020). New insight into sortilin in controlling lipid metabolism and the risk of atherogenesis. Biol. Rev. Camb. Philos. Soc..

[30] Ren L., Sun Y., Lu H., Ye D., Han L., Wang N., Daugherty A., Li F., Wang M., Su F. (2018). (Pro)renin Receptor Inhibition Reprograms Hepatic Lipid Metabolism and Protects Mice From Diet-Induced Obesity and Hepatosteatosis. Circ. Res..

[31] Morimoto S., Morishima N., Watanabe D., Kato Y., Shibata N., Ichihara A. (2021). Immunohistochemistry for (Pro)renin Receptor in Humans. Int. J. Endocrinol..

[32] Worker C.J., Li W., Feng C.Y., Souza L.A.C., Gayban A.J.B., Cooper S.G., Afrin S., Romanick S., Ferguson B.S., Feng Earley Y. (2020). The neuronal (pro)renin receptor and astrocyte inflammation in the central regulation of blood pressure and blood glucose in mice fed a high-fat diet. Am. J. Physiol. Endocrinol. Metab..

[33] Bokuda K., Ichihara A. (2014). Possible contribution of (pro)renin receptor to development of gestational diabetes mellitus. World J. Diabetes.

[34] Wang F., Luo R., Zou C.J., Xie S., Peng K., Zhao L., Yang K.T., Xu C., Yang T. (2020). Soluble (pro)renin receptor treats metabolic syndrome in mice with diet-induced obesity via interaction with PPARγ. JCI Insight.

[35] Holst J.J. (2019). From the Incretin Concept and the Discovery of GLP-1 to Today’s Diabetes Therapy. Front. Endocrinol..

[36] Ramkumar N., Kohan D.E. (2019). The (pro)renin receptor: An emerging player in hypertension and metabolic syndrome. Kidney Int..

[37] Xu C., Liu C., Xiong J., Yu J. (2022). Cardiovascular aspects of the (pro)renin receptor: Function and significance. FASEB J..

[38] Qu X., Harmelink C., Baldwin H.S. (2022). Endocardial-Myocardial Interactions During Early Cardiac Differentiation and Trabeculation. Front. Cardiovasc. Med..

[39] Smiljic S. (2017). The clinical significance of endocardial endothelial dysfunction. Medicina.

[40] Xu Q., Jensen D.D., Peng H., Feng Y. (2016). The critical role of the central nervous system (pro)renin receptor in regulating systemic blood pressure. Pharmacol. Ther..

